# Pre- and post-implementation protocol for non-operative management of grade III-V splenic injuries: An observational study

**DOI:** 10.1016/j.heliyon.2024.e28447

**Published:** 2024-03-20

**Authors:** Ismail Mahmood, Basil Younis, Mohammad Alabdallat, Saji Mathradikkal, Husham Abdelrahman, Ayman El-Menyar, Mohammad Asim, Mohammad Kasim, Monira Mollazehi, Ammar Al-Hassani, Ruben Peralta, Sandro Rizoli, Hassan Al-Thani

**Affiliations:** a Department of Surgery, Trauma Surgery Section, Hamad Medical Corporation (HMC), Doha, Qatar; b Department of Surgery, Trauma and Vascular Surgery, Clinical Research, HMC, Doha, Qatar; c Clinical Medicine, Weill Cornell Medical College, Doha, Qatar; d Department of Surgery, Trauma Surgery, National Trauma Registry, HMC, Doha, Qatar; e Department of Surgery, Trauma and Vascular Surgery, HMC, Doha, Qatar

**Keywords:** Blunt abdominal trauma, Non-operative management, Blunt splenic injury, Splenic arterial embolization

## Abstract

**Background:**

Grade (III–V) blunt splenic injuries (BSI) in hemodynamically stable patients represent clinical challenges for successful non-operative management (NOM). In 2014, Our institution proposed a treatment protocol requiring splenic angiography and embolization for stable, intermediate, and high-grade BSI. It also included a follow-up CT scan for grade III BSI. We sought to assess the success rate of NOM in treating intermediate and high-grade BSI, following a standardized treatment protocol at a level 1 trauma center.

**Methods:**

An observational retrospective study was conducted. Data of patients with BSI from June 2011 to September 2019 were reviewed using the Qatar National Trauma Registry. Patients’ demographics, CT scan and angiographic findings, grade of splenic injuries, and outcomes were analyzed. The pre- and post-implementation of treatment protocol periods were compared.

**Results:**

During the study period, a total of 552 hemodynamically stable patients with BSI were admitted, of which 240 had BSI with grade III to V. Eighty-one patients (33.8%) were admitted in the pre-protocol implementation period and 159 (66.2%) in the post-protocol implementation period. The NOM rate increased from 50.6% in the pre-protocol group to 65.6% in the post-protocol group (p = 0.02). In addition, failure of the conservative treatment did not significantly differ in the two periods, while the requirement for blood transfusion dropped from 64.2% to 45.9% (p = 0.007). The frequency of CT scan follow-up (55.3% vs. 16.3%, p = 0.001) and splenic arterial embolization (32.7% vs. 2.5%, p = 0.001) in NOM patients increased significantly in the post-protocol group compared to the pre-protocol group. Overall mortality was similar between the two periods. However, hospital and ICU length of stay and ventilatory days were higher in the post-protocol group.

**Conclusions:**

NOM is an effective and safe treatment option for grade III-V BSI patients. Using standardized treatment guidelines for intermediate-to high-grade splenic injuries could increase the success rate for NOM and limit unnecessary laparotomy. Moreover, angioembolization is a crucial adjunct to NOM that could improve the success rate.

## Highlights

• The management of Grade III-V Splenic Injury remains a clinical challenge for trauma surgeons.
• The utility of standardized treatment protocol for moderate to severe splenic injuries is effective and safe.
• This protocolized approach resulted in improved non-operative management rates from 50.6% to 65.6%.
• The protocol decreased the surgical intervention from 49.4% to 34.6%, and blood transfusion from 64.2% to 45.9%.
• However, as with any treatment modality, the cost and risk of complications must be considered.

## 1 Introduction

The spleen is one of the most frequently injured intra-abdominal solid organs, which is affected in one-third of patients with blunt abdominal trauma (BAT) [1,2]. Blunt splenic injury (BSI) requires prompt diagnosis and management to avoid potentially life-threatening bleeding in severely injured patients [3,4]. In Qatar, the BSI is mainly caused by traffic-related trauma (70.5%), and it represents about (22%) of all BAT [5], which requires protocolized management. During the last few decades, the inception of high-resolution diagnostic imaging and angiography has facilitated the evolving landscape of BSI management towards non-operative management (NOM) [6].

For patients who are hemodynamically stable and have no peritoneal symptoms, NOM is the preferred treatment approach, with a lesser failure rate (4–13%), even in those who presented with high-grade BSI (IV or V) [[7], [8], [9]]. Also, NOM preserves the immunologic function of the spleen, requires lesser blood transfusion, and has a lower rate of complications and mortality compared to operative management (OM) [10,11]. Moreover, angioembolization (AE) is a valuable adjunct to the NOM, with a higher rate of splenic salvage (80–98%) [8]. According to the American Association for the Surgery of Trauma–Organ Injury Score (AAST-OIS) [9], AE should be considered in patients with grade III or higher BSI.

With the technological advances in diagnostic imaging and the role of AE being more defined, an increasing number of trauma centers worldwide have begun to adopt a protocolized approach incorporating AE in the management of stable patients with high-grade BSI. Consequently, this has led to higher rates of spleen salvage and lesser NOM failure [12]. However, whether NOM is effective for high-grade BSI remains to be addressed. Considering the evidence-based management practice [13,14], our institution implemented guidelines for managing hemodynamically stable patients with traumatic splenic injuries in 2014. The present study aims to evaluate the effectiveness of the new protocolized management guidelines for the BSI (2015–2019) in comparison to the earlier cohort (2011–2014) at a level-I trauma center in Hamad General Hospital (HGH). We hypothesize that these new guidelines (protocol) would improve the institution's success rate of NOM in patients with intermediate to high-grade (III–V) BSI.

## 2 Methods

This was a retrospective observational study to assess the data-driven performance improvement at a national Level I trauma center, Hamad Trauma Center (HTC), Doha, Qatar. Data were retrieved from the Qatar National Trauma Registry (QNTR) for all patients with BSI admitted at Hamad Level I Trauma Center between June 2011 and September 2019. The QNTR has internal and external validation and is linked to the National Trauma Database Bank (NTDB) and the American College of Surgeons Trauma Quality Improvement Program (ACS-TQIP) [15,16]. All adult patients (age ≥14 years) with grade III-V BSI were included in the study. Pediatric patients (age <14 years) with splenic injuries from penetrating trauma, iatrogenic splenic injuries, and hemodynamically unstable BSI patients taken to the operating room without a CT scan were excluded. The hemodynamically stable patients with BSI were considered for NOM.

Data included demographic characteristics, mechanism of injury, initial vital signs, associated injuries, Injury Severity Score, Glasgow Coma Score on admission, grade of splenic injury (III–V), presence or absence of contrast blush on initial CT scan, repeat CT scan findings, management approach (NOM or operative), failure of NOM, indications for AE, splenic arterial embolization (SAE), and technique of angioembolization (proximal, distal or combined), failure of angioembolization, hospital course and mortality. The splenic injuries were graded according to the American Association for the Surgery of Trauma (AAST) Organ injury [9]. The study's primary outcome measure was the success rate of NOM and angioembolization.

Failure of NOM was considered during observation in case of hemodynamic instability, progressive drop in hemoglobin level despite angioembolization, diffuse peritonitis, or detection of missed abdominal injuries requiring surgery. NOM was considered successful when the patient was discharged with intact spleen in the abdominal cavity. Pre-protocol refers to the pre-implementation duration (2011–2014) of the standard guideline for BSI management. In contrast, the protocol period corresponds to the post-implementation period (2015–2018) of the BSI management guidelines at our institution. The hemodynamically unstable patients were considered if systolic blood pressure was less than 90 mmHg, heart rate was over 100 bpm, and not stabilized after standard fluid resuscitation.

**Institutional BSI management guidelines:** Before June 2014, patients with contrast blush shown on their admission CT scan underwent angiography. Moreover, embolization was carried out at the angiographer's discretion, frequently because of identifying pseudoaneurysms or other vascular injuries during angiography. Based on the findings of the published literature [13,14], our department started a process improvement project in 2014 to manage hemodynamically stable patients with BSI. The new guidelines (protocol) of BSI management depend on the hemodynamic status of the patient and the grade of splenic injury in accordance with the American Association of Surgery of Trauma Organ Injury Score (AAST-OIS), which divides the splenic injury into five grades (I and II as low grades, III as intermediate grade, and IV and V as high grades). Splenic salvage with NOM is considered in hemodynamically stable patients with low-grade BSI (I and II), whereas splenectomy is indicated in hemodynamically unstable patients. Additionally, angiography and embolization are performed for stable patients presenting with grades IV-V BSI. For hemodynamically stable Grade III injuries by CT scan with no evidence of contrast extravasation, a follow-up CT scan within 72 h of admission is needed, and an intervention radiology angiographic evaluation and embolism is indicated for moderate to large hemoperitoneum.

BSI patients with lower-grade injuries were also referred for angiography if they displayed a contrast blush on their admission CT scan. The decision to perform the type of embolization, namely, proximal splenic artery, distal selective embolization, or both, was based on the interventional radiologist's discretion, based on anatomical findings, and available technology at the time of the study. Additionally, in case of clinical disagreement among the intervention radiologists as to whether embolization should be performed on all patients or only on those who had been found to have a definite vascular injury on angiography. Therefore, it was up to each clinician to decide whether to continue embolization. Contrast-enhanced computed tomography scanners were performed using Siemens Medical Systems (Siemens, Erlangen, Germany). Scans were performed after injecting 120 mL of iohexol (Omnipaque, GE Healthcare, Waukesha, WI) at 3 mL/s. CT scan Images through the abdomen were reconstructed at 1.5-mm, 2.5-mm, and 5-mm slice thickness. Pre- and post-contrast scans were usually performed. A consultant trauma radiologist assessed the images.

The institutional medical review board at the Medical Research Center (MRC) at Hamad Medical Corporation, Doha, Qatar (Protocol no: MRC-01-19-481) approved the study protocol. A waiver of consent was granted as data were collected retrospectively and anonymously without direct contact with patients.

**Statistical Analysis**: Data were presented as proportions, medians (interquartile range; IQR), or mean (± standard deviation; SD) as appropriate. The variables of interest were compared and analyzed according to the pre-protocol and protocol implementation of the institutional BSI management guidelines. Differences in categorical and continuous variables were analyzed using the χ2 and student-t tests, as appropriate. Fisher's exact test was used for categorical variables if the cell value was less than five. The Mann–Whitney *U* test was used for non-parametric data whenever applicable. A significant difference was considered when the 2-tailed p-value was less than 0.05. Data analysis was carried out using the Statistical Package for Social Sciences version 28 (SPSS Inc., Chicago, IL).

## 3 Results

During the study period, 552 hemodynamically stable patients with BSI were admitted to our institution, of which 312 had low-grade (1 and II) BSI and were excluded. Finally, 240 BSI cases with grades III to V were included in the analysis, of which 81 (33.8%) were admitted in the pre-protocol implementation, and 159 (66.2%) were admitted in the post-protocol implementation group. Table 1 shows the demographic and clinical variables for patients with grade III-V BSI in the pre-protocol and protocol groups. The two groups did not differ significantly concerning age, gender, mechanism of injury, initial vitals, injury severity score, Glasgow coma score, abdominal AIS, and associated head injury. Chest injuries such as rib fracture (65.4% vs. 48.1%, p = 0.01), pneumothorax (32.7% vs. 18.5%, p = 0.02), and hemothorax (18.9% vs. 8.6%, p = 0.03) were higher in the pre-protocol group. Still, the rate of other solid organ injuries involving the liver, kidney, pancreas, and bowel/mesentery were similar between the two periods.

**Table 1:** Demographic and clinical characteristics stratified by treatment protocol period in patients diagnosed with grade III-V Blunt Splenic Injuries (BSI).

Variables	Pre-protocol^a^ (n = 81)	protocol** (n = 159)	*P*-value^
**Age (mean ± SD) (n** = **237)**	28.1 ± 10.2	30.9 ± 11.1	0.05
**Males**	75 (92.6%)	144 (90.6%)	0.59
**Mechanism of Injury**			
Traffic related	53 (66.3%)	111 (69.8%)	0.49 for all
Fall from height	18 (22.5%)	28 (17.6%)	
Fall of heavy object	2 (2.5%)	1 (0.6%)	
Others	8 (9.9%)	19 (11.9%)	
**Systolic blood pressure (n** = **231)**	106.8 ± 30.7	115.4 ± 22.8	0.01
**SBP<100**	21 (26.9%)	27 (17.6%)	0.10
**Diastolic blood pressure (n** = **226)**	69.4 ± 16.9	72.7 ± 16.9	0.16
**FAST positive**	48 (59.3%)	112 (70.4%)	0.08
**Associated injuries**			
Head injury	23 (28.4%)	53 (33.3%)	0.43
Rib fracture	39 (48.1%)	104 (65.4%)	0.01
Lung contusion	33 (40.7%)	69 (43.4%)	0.69
Pneumothorax	15 (18.5%)	52 (32.7%)	0.02
Hemothorax	7 (8.6%)	30 (18.9%)	0.03
**Other solid organ injuries**			
Hepatic	15 (18.5%)	28 (17.6%)	0.86
Kidney	13 (16.0%)	21 (13.2%)	0.55
Pancreas	7 (8.6%)	9 (5.7%)	0.38
Bowel/mesenteric	5 (6.2%)	18 (11.3%)	0.20
**Injury Severity Score**	26.3 ± 11.5	28.4 ± 11.8	0.17
**Chest AIS (n** = **186)**	2.96 ± 0.78	2.87 ± 0.71	0.42
**GCS ED (median, 95% CI) (n** = **235)**	15 (11.2–13.3)	15 (11.3–12.9)	0.85
**Abdominal AIS**	3.5 ± 1.1	3.6 ± 0.9	0.38

a Pre-implementation i.e. before standard guideline for BSI management (2011–2014); ** Post-implementation i.e. after implementation of institutional BSI management guidelines (2015–2018); AIS: Abbreviated Injury Scale; GCS: Glasgow Coma Score at emergency department; ^Differences in categorical and continuous variables were analyzed using the χ2 test and students t-test, as appropriate to compare the early versus late BSI management groups. Also, Fisher's exact test was used for categorical variables, if the cell value was less than 5. Also, Mann–Whitney *U* test was used for non-parametric data; whenever applicable.

Table 2 compares the radiological and intra-operative findings, management, and outcome in the pre-protocol and protocol groups. Based on the initial CT scan findings, contrast blush on CT scan (36.5% vs. 19.8%, p = 0.008) and pseudoaneurysm (17.6% vs. 2.5%, p = 0.001) were more likely to be identified in the protocol group. The frequency of shattered spleen and devascularization was comparable among the two groups. As expected, the frequency of follow-up CT scan (55.3% vs. 16.3%, p = 0.001) and splenic arterial embolization (32.7% vs. 2.5%, p = 0.001) in NOM patients increased significantly in the protocol group, in comparison to the pre-protocol group. A follow-up CT scan detected pseudoaneurysms in 12 patients; nine patients were embolized, two patients were treated conservatively as the size of the pseudoaneurysm was less than 5 mm, and one patient refused intervention.

**Table 2:** Comparative analysis of radiological and intra-operative findings, management approaches, and outcome between the pre and protocol periods.

Variables	Pre-protocol^a^ (n = 81)	Protocol** (n = 159)	P value^
Initial CT scan findings of spleen			
Contusion	46 (56.8%)	45 (28.3%)	0.001
Laceration	39 (48.1%)	144 (90.6%)	0.001
Blush	16 (19.8%)	58 (36.5%)	0.008
Shattered spleen	18 (22.2%)	24 (15.1%)	0.16
**Revascularization**	1 (1.2%)	8 (5.0%)	0.14
Pseudoaneurysm	2 (2.5%)	28 (17.6%)	0.001
**Repeat CT scan**			
Done	13 (16.3%)	88 (55.3%)	0.001 for all
Not done due to splenectomy	43 (53.8%)	60 (37.7%)	
Not done	25 (30.9%)	11 (6.9%)	
**Splenic injury grades**			
III	44 (54.3%)	74 (46.5%)	0.001 for all
IV	17 (21.0%)	74 (46.5%)	
V	20 (24.7%)	11 (6.9%)	
**Blood transfusion**	52 (64.2%)	73 (45.9%)	0.007
**Treatment**			
NOM	41 (50.6%)	109 (68.6%)	0.007 for all
Operative management	40 (49.4%)	50 (31.4%)	
**Failure of NOM (n** = **5)**	1 (1.2%)	4 (2.5%)	0.51
**Splenic arterial embolization (n** = **54)**	2 (2.5%)	52 (32.7%)	0.001
**Type of embolization**			
Proximal	0 (0.0%)	27 (51.9%)	0.24 for all
Distal	2 (100%)	21 (40.4%)	
Combined	0 (0.0%)	4 (7.7%)	
**Failure of embolization (n** = **3)**	0 (0.0%)	3 (1.9%)	0.21
**Hospital length of stay (days)**	6 (1–304)	10 (1–127)	0.001
**ICU length of stay (days)**	3 (1–37)	4 (1–60)	0.04
**Ventilatory days**	1 (1–27)	6 (1–47)	0.007
**Mortality**	10 (12.3%)	18 (11.3%)	0.81

a Pre-implementation i.e. before standard guideline for BSI management (2011–2014); ** Post-implementation i.e. after implementation of institutional BSI management guidelines (2015–2018); CT: computed tomography; NOM: non operative management; ^ Differences in categorical and continuous variables were analyzed using the χ2 test and students t-test, as appropriate to compare the early versus late BSI management groups. Also, Fisher's exact test was used for categorical variables, if the cell value was less than 5. Also, Mann–Whitney *U* test was used for non-parametric data; whenever applicable.

Overall, patients in the protocol group were more likely to have higher grade IV BSI, whereas grades III and V were more frequently observed in the pre-protocol group (p = 0.001).

**Fig. 1:**
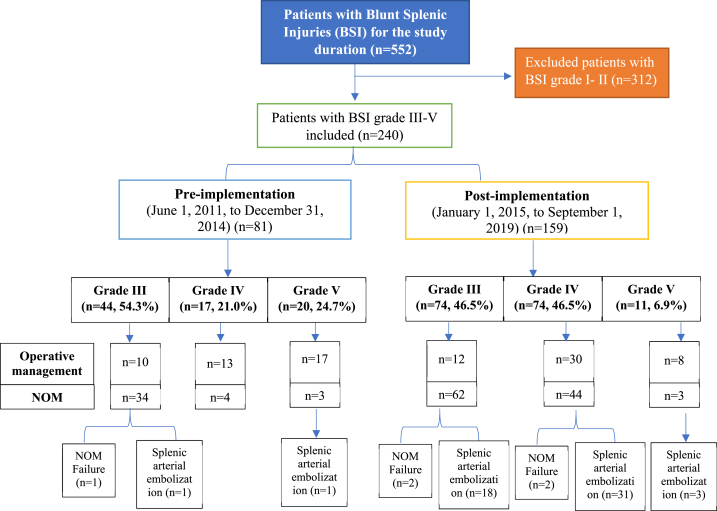
Study flow chart.

**Fig. 2:**
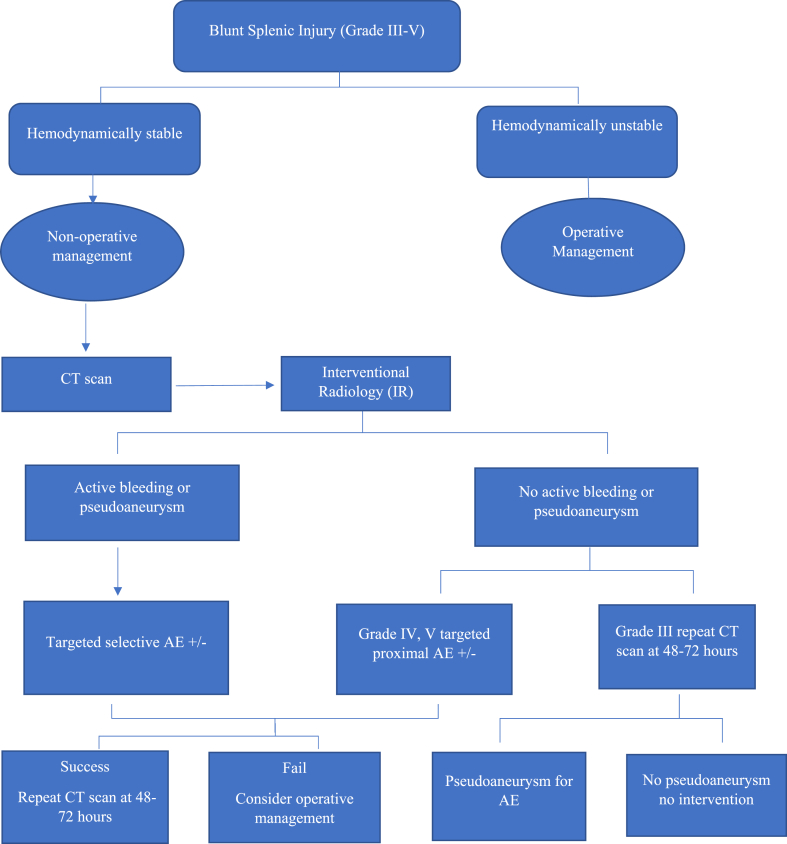
Algorithm of blunt splenic injuries (BSI) management for grade III-V

Fig. 1 shows the study design. In the pre-protocol period, 44 patients (54.3%) with grade III injuries were seen, of which 34 (77.3%) underwent NOM. Only one patient experienced NOM failure and required splenectomy. In the protocol group, 74 patients with grade III injuries were admitted, of which 62 (83.8%) had NOM, only two patients had a failure of NOM, and 18 were managed by splenic arterial embolization. Concerning grade IV BSI, 17 cases were observed in the pre-protocol group, and 74 were in the protocol group. In the pre-protocol and protocol groups, NOM was used in 4 (23.5%) and 44 (59.5%) patients, respectively. There was no failure of NOM in the pre-protocol group, whereas 2 failed NOM in the protocol group. Also, 31 patients underwent splenic arterial embolization in the protocol group as opposed to none in the pre-protocol group. Injuries of Grade V occurred less frequently in the protocol group (6.9%) than in the pre-protocol group (24.7%). Only 15% (n = 3) and 27.3% (n = 3) of patients in pre-protocol and protocol groups had NOM, respectively, and there were no treatment failures in either group. In the protocol group, 27 patients (51.9%) were treated by proximal embolization, 21 (40.4%) by distal embolization, and 4 (7.7%) by combined embolization. Fig. 2 shows the BSI management algorithm used for Grade III-V patients. However, in the pre-protocol group, only two patients had distal embolization (Table 2).

In the pre-protocol group, the initial management was urgent splenectomy in 40 patients (50.6%) compared to 50 patients (31.5%) in the protocol group. The main indication for surgery was hemodynamic instability, and an abdominal CT scan was performed as part of the secondary survey at admission. The NOM rates increased from 50.6% in the pre-protocol group to 68.6% in the protocol group (P = 0.007), and operative management decreased from 49.4% to 31.6% (p = 0.007). In addition, failure in conservative treatment was not significant in the two groups, and the requirement for blood transfusion dropped from 64.2% of the patients to 45.9% in the protocol group (P = 0.007). Overall, the use of splenic arterial embolization in NOM patients increased significantly in the protocol period (32.7% vs. 2.5%, p = 0.001) than in the pre-protocol period. Examining NOM failures, failed hemostasis with recurrent bleeding was found in five patients. Two had angiography with embolization, two patients underwent splenic artery embolization, and one underwent distal selective embolization. In addition, one patient underwent angiography without embolization, and the remaining two had no angiography.

Table 3 shows the clinical characteristics and splenic injury grades of patients who experienced non-operative management (NOM) failure.

**Table 3:** Clinical characteristics and splenic injury grades of patients who experienced non-operative management (NOM) failure.

Cases	Age (years)	Injury details and cause of NOM failure	Splenic injury grade	Time of failure
**Patient 1**	24	Fracture of upper end plate of T 8 and T 7 vertebra, deep laceration at the lateral side of the left thigh. Failure of proximal splenic artery embolization. Hemodynamic instability. Active bleeding from splenic parenchyma laceration.	Grade 5	One day
**Patient 2**	33	Right iliac bone fracture. Grade 2 liver injury. No angiogram. Persistent drop in hemoglobin level. Large hemoperitoneum.	Grade 4	4 days
**Patient 3**	31	Fracture of the left first, fifth and eight ribs. Fractures of the left superior and inferior pubic rami. Bilateral sacral ala fractures. Splenic artery angiography revealed no evidence of active bleeding or pseudo-aneurysm formation. No embolization was performed (angiographer decision). Persistent drop in hemoglobin level. Large hemoperitoneum.	Grade 3	3 days
**Patient 4**	60	Fractures of the second, third, fourth fifth sixth and seventh ribs on the left side Intimal aortic injury Failure of combined proximal and distal splenic embolization Persistent fall in hemoglobin Large hemoperitoneum	Grade 3	6 days
**Patient 5**	37	Fractures left eight, nine, ten and eleven ribs. Left adrenal gland injury/hematoma. Failure of proximal splenic embolization. Hemodynamic instability. Bleeding from short gastric vessels.	Grade 4	3 days

The average time elapsed between admission to the delayed splenectomy was 64 h (range 6–96 h). Indications for delayed splenectomies were a persistent fall in hemoglobin in four patients and hemodynamic instability in the other two patients. Two significant complications were observed: one patient developed a splenic abscess, which required percutaneous drainage, and another developed a splenic cyst, for which robotic splenectomy was performed.

The hospital course regarding ventilatory days, ICU days, and hospital length of stay were higher in the protocol group (P = 0.007). However, the overall mortality rate was comparable between the two periods, and the cause of the deaths was not related to the BSI per se but mainly to the associated injury.

## 4 Discussion

The present study evaluated the implementation of an institutional protocol for managing intermediate to high-grade (III–V) BSIs to improve the outcome of NOM in 240 hemodynamically stable patients admitted to a level 1 trauma center in a Middle Eastern country. Adopting a protocol for the treatment of trauma patients would decrease the communication gaps among the treating team and save time, resources, and patient's lives. This study has several important findings. We found that, in contrast to the operative management option, which decreased from 49.4% to 31.6%, the rate of NOM significantly increased from 50.6% in the pre-protocol period to 68.6% in the protocol implementation period. Moreover, increasing the use of follow-up CT scans and splenic arterial embolization in the protocol period led to the success of NOM in grade III-V BSI patients who were managed according to the standard guidelines.

Recent clinical data showed an increase in the success rate of NOM of BSI. Over seven years, Aoki et al. [17] demonstrated an increasing trend in the adoption of NOM for high-grade BSIs among hemodynamically stable patients, with a substantial drop in the splenectomy rate from 22.9% to 12.6% and a significant increase in the utilization of SAE from 12.5% to 20.9%. It has been reported that using angioembolization according to the protocol in patients with higher-grade (III to V) injuries leads to a better success rate of NOM, reaching as high as 95% [[17], [18], [19]]. Clinical practice has improved because of various therapeutic options, including repeat CT scans, selective or main splenic angioembolization, and ongoing surveillance [20]. Among these, splenic angioembolization has significantly increased the likelihood of successful NOM in BSI [12,13,21]. However, the ideal application of this procedure remains controversial [[21], [22]]. However, the risk of splenic infarction, fever, pleural effusion, splenic abscess, contrast-induced renal failure, and splenic bleeding are examples of the complications that are associated with SAE, which may occur in up to 47% of cases [23,24].

The present study shows that the frequency of SAE (2.5%–35.2%) and follow-up CT scans (16.3%–63.5%) has significantly increased at our institution following the new guidelines. Several factors might contribute to this accomplishment. Firstly, our trauma center is the only Level 1 facility in Qatar with preprogrammed responses incorporated into patient referral and treatment protocols with an efficient ground and air ambulance with a 24-h helipad. This has undoubtedly helped reduce mortality and the time required for definitive bleeding control. Furthermore, a multidisciplinary trauma team strategy to damage control resuscitation upon arrival at the trauma center has reduced the time to stabilization, access to CT scan, and resuscitation. This may allow opting for NOM intervention rather than immediate operative intervention. The increasing trend also reflects the growing body of evidence supporting the role of SAE in assisting NOM in severe blunt trauma [25]. In clinical practice, the decision between NOM and operative management is mainly guided by the hemodynamic status rather than the severity of organ damage [12,26]. While the advantages of NOM are undeniable, there is still a lack of agreement about patient stratification and possible risk factors associated with the failure of NOM.

The current study showed that the NOM, carried out following our recommendations, is a secure and reliable option for treating patients with high-grade BSI. Indeed, the total success rate of NOM was about 96% for all grades of splenic injuries, with no risk of mortality or significant complications. In line with our findings, the literature has frequently reported that spleen-preserving treatment by NOM is safe and effective for low-grade and high-grade injuries [14,18,[27], [28], [29]]. Implementing the guideline for moderate-to-severe BSI at our institution has improved the NOM rate from 50.6% to 65.6% and reduced surgical intervention from 49.4% to 34.6%. These results are consistent with earlier studies [12,21,29].

A multicenter study [30], including 27 trauma centers in the US, reported a success rate of NOM to be 61.5%. However, the failure rate was 10.8%, primarily associated with compromised vital signs on admission. In patients who initially underwent NOM, the failure rate increased significantly by the BSI grades, i.e., Grade I (4.8 %), II (9.5%), III (19.6%), IV (33.3%), and V (75.0%). In a subgroup with unsuccessful NOM, 60% of deaths were caused by delayed treatment of splenic or other abdominal injuries [31].

An earlier study by Miller et al. [18] developed a protocol requiring the referral of all stable patients with grade III -V BSI for angiography and embolization. There was an improvement in the success rate of NOM after adapting the protocol (67%) compared to the historical control (52%) group. Moreover, the protocol used in stable, high-grade BSI patients led to a significantly lower failure rate. Similarly, Brillantino et al. [12] adopted a similar protocol for 87 patients with CT findings of vascular injuries and those with steadily declining hemoglobin levels despite the absence of vascular injuries on the CT scan. The authors reported a higher success rate of NOM in grade III (95%), grade IV (90.9%), and grade V (83.0%) BSI.

In our study, IR was performed in more than one-third of patients, and only 1.9% had a failure of embolization. Therefore, the risk of procedure-related complications should be balanced against the risks of unnecessary trauma laparotomies and long-term immune deficiency, which can lead to fulminant infections. In addition, a dedicated IR on-call team allows rapid activation of services. The standardized protocol aids in the decision-making process for managing patients with BSI is outlined in Fig. 2. Consistent with our findings, angioembolization has been recommended in earlier studies for higher-grade injuries and those where vascular injury is visible on a CT scan [32,33]. Chen et al. suggested that, in higher-grade injuries, angioembolization is a crucial adjunct to NOM that improves NOM success [34]. Contrarily, an earlier study showed that splenic artery angiography and embolization did not affect the success rate of NOM in patients with BSI [35]. Also, in our study, the protocol group showed prolonged hospital length of stay and increased ventilatory days. These findings are likely attributable to the nature of conservative management, severe overall injuries, and higher occurrence of associated injuries, such as rib fractures, pneumothoraces, and mesothoraces. Previous studies have also reported substantial hemoperitoneum and splenic infarction, which could contribute to these prolonged hospital stays and the need for mechanical ventilation [36,37]. In addition, complications, such as splenic infarction, can result in a significant delay in hospital discharge compared to patients who had splenectomy. A similar result was reported by Duchesne et al. [24], who reported an increased incidence of respiratory complications such as acute respiratory distress syndrome in a group of patients managed by angiographic embolization compared with those who were treated by splenectomy.

Limitations of the study. The retrospective study design and experience with a single trauma center are drawbacks that may result in missing data and affect the findings' generalizability. Secondly, information regarding follow-up post-discharge should have been recorded systematically and would be of interest in further prospective studies. Also, we need more information pertaining to the infection, procedure-related morbidity, and complications, such as splenic infarction rate and systematic use of interventional radiology, that might be beneficial in validating these findings and improving the quality of patient care. Besides systematically re-evaluating radiological reports and performing splenic arterial angioembolization, it outlines significant data on injury patterns, surgical and NOM details, and outcome metrics following new guidelines at our center and improves knowledge and learning curve in this specific subject. A larger sample size would be suitable for propensity-matched analysis between the study groups and more solid results.

Angioembolization was practiced in our institute before December 2014; thus, implementing a protocol has increased the number of angioembolizations from 2.5% to 32.7% while reducing the need for trauma laparotomy (operative management) from (1 of 2) of high-grade splenic injuries to 1 of 3), without affecting mortality. In addition, the angioembolization protocol also reduced the need for blood transfusion. Lastly, the age cut-off in our institute for adult subjects is 14 and above. Still, there is a controversy in defining age groups in the literature [38].

## 5 Conclusions

Non-operative management is an effective and safe treatment option for patients with grade III-V blunt splenic injuries following a management algorithm. Using guidelines for intermediate and high-grade splenic injuries could increase the success rate for NOM and limit unnecessary laparotomy. Moreover, angioembolization is a crucial adjunct to NOM. However, liberal use of angioembolization has been associated with increased ventilatory days, ICU days, and hospital length of stay. So, it is recommended that trauma centers implement algorithms or institutional evidence-based guidelines for BSI management that are compatible with safe practice and local resources. Furthermore, larger prospective studies might be beneficial in validating these findings and improving patient care.

## Funding

none.

## Data and material availability

All data are given in the manuscript, table, and figure. Data associated with this study will be available from the corresponding author upon reasonable request and after approval of the Medical Research Center at Hamad Medical Corporation and signing a data sharing agreement form.

## CRediT authorship contribution statement

**Ismail Mahmood:** Writing – original draft, Methodology, Data curation, Conceptualization. **Basil Younis:** Data curation, Conceptualization. **Mohammad Alabdallat:** Data curation, Conceptualization. **Saji Mathradikkal:** Data curation, Conceptualization. **Husham Abdelrahman:** Methodology, Data curation, Conceptualization. **Ayman El-Menyar:** Writing – review & editing, Writing – original draft, Formal analysis, Conceptualization. **Mohammad Asim:** Writing – original draft, Formal analysis. **Mohammad Kasim:** Data curation, Conceptualization. **Monira Mollazehi:** Data curation. **Ammar Al-Hassani:** Writing – review & editing, Methodology, Conceptualization. **Ruben Peralta:** Writing – review & editing, Conceptualization. **Sandro Rizoli:** Writing – review & editing, Supervision, Conceptualization. **Hassan Al-Thani:** Writing – review & editing, Supervision, Conceptualization.

## Declaration of competing interest

The authors declare that they have no known competing financial interests or personal relationships that could have appeared to influence the work reported in this paper.

## List of abbreviations

BSI: blunt splenic injury
BAT: blunt abdominal trauma
ISS: injury severity score
NOM: non-operative management
OM: operative management
AE: angioembolization
SAE: splenic arterial angioembolization
IR: interventional radiology
ICU: Intensive care unit
AAST-OIS: American Association for the Surgery of Trauma–Organ Injury Score
ACS TQIP: American College of Surgeons Trauma Quality Improvement Program
HTC: Hamad Trauma Center
QNTR: Qatar National Trauma Registry
NTDB: National Trauma Database Bank
